# Vascular Metabolism as Driver of Atherosclerosis: Linking Endothelial Metabolism to Inflammation

**DOI:** 10.20900/immunometab20210020

**Published:** 2021-05-17

**Authors:** Kim E. Dzobo, Katie M. L. Hanford, Jeffrey Kroon

**Affiliations:** Amsterdam UMC, University of Amsterdam, Department of Experimental Vascular Medicine, Amsterdam Cardiovascular Sciences, Meibergdreef 9, Amsterdam, 1105 AZ, The Netherlands

**Keywords:** atherosclerosis, endothelial metabolism, inflammation

## Abstract

The endothelium is a crucial regulator of vascular homeostasis by controlling barrier integrity as well acting as an important signal transducer, thereby illustrating that endothelial cells are not inert cells. In the context of atherosclerosis, this barrier function is impaired and endothelial cells become activated, resulting in the upregulation of adhesion molecules, secretion of cytokines and chemokines and internalization of integrins. Finally, this leads to increased vessel permeability, thereby facilitating leukocyte extravasation as well as fostering a pro-inflammatory environment. Additionally, activated endothelial cells can form migrating tip cells and proliferative stalk cells, resulting in the formation of new blood vessels. Emerging evidence has accumulated indicating that cellular metabolism is crucial in fueling these pro-atherosclerotic processes, including neovascularization and inflammation, thereby contributing to plaque progression and altering plaque stability. Therefore, further research is necessary to unravel the complex mechanisms underlying endothelial cell metabolic changes, and exploit this knowledge for finding and developing potential future therapeutic strategies. In this review we discuss the metabolic alterations endothelial cells undergo in the context of inflammation and atherosclerosis and how this relates to changes in endothelial functioning. Finally, we will describe several metabolic targets that are currently being used for therapeutic interventions.

## Introduction

The endothelium is a crucial barrier between blood and tissue and is essential for maintaining vascular homeostasis [1]. The monolayer of endothelial cells (ECs) covering the vascular wall are exposed to several mechanical (stretch, shear stress, pressure) and circulating factors (cytokines, chemokines, humoral agents, chemical factors, lipoproteins) that can all affect their phenotype. Throughout vascular homeostasis ECs are in a quiescent state, characterized by the formation of nitric oxide (NO) by endothelial nitric oxide synthase (eNOS). NO has been considered to be atheroprotective due to its anti-inflammatory role by regulating vasodilatation, inhibiting thrombosis and the adhesion of leukocytes and platelets [2]. However, when ECs are exposed to disturbed or low flow conditions, they exhibit a loss in eNOS activity and an enhanced activated phenotype [3]. This EC activation can result in (1) upregulation of adhesion molecules involved in efficient leukocyte capturing and extravasation, (2) internalization of the junctional protein VE-cadherin; resulting in increased vascular permeability, (3) activated ECs secreting cytokines and chemokines; promoting inflammation and involved in attracting (more) immune cells to the site of infection [3–6]. Furthermore, during the advanced stages in atherosclerosis, the hypoxic regions in atherosclerotic plaques can lead to local production of the pro-angiogenic factor VEGF, resulting in plaque neovascularization [7–9]. Overall, these processes can contribute to further progression of atherosclerosis, thereby aggravating clinical outcome.

To sustain these pro-atherogenic processes, a certain amount of energy and biomass is necessary. In the field of cancer biology, rewiring of cellular metabolism has been extensively explored as a way for cancer cells to gain a substantial amount of energy and biomass required for proliferation, invasion and metastasis [10,11]. Cancer cells switch from oxidative phosphorylation to aerobic glycolysis for the production of ATP [12,13]. This metabolic switch is referred to as the Warburg effect. Interestingly, this rewiring of cellular metabolism has also been observed during atherosclerosis, which has predominantly been described in macrophages [14,15]. Recently, it has been established that vulnerable human atherosclerotic lesions exhibit an enhanced expression of glycolytic markers compared to stable plaques [16,17]. Several landmark studies of the group of Carmeliet have shown that ECs are highly glycolytic, a phenomenon which is also the case under quiescent conditions [18].

However, to date the role of EC metabolism in atherosclerosis has been studied to a lesser extent. In this review we will discuss the intricate role of EC metabolism in fueling vascular inflammation and atherogenesis. Lastly, we will address various signaling routes targeting microRNA-124, the mitochondria, the glycolytic enzyme 6-phosphofructo-2-kinase/fructose-2,6-biphosphatase 3 (PFKFB3), lipoprotein (a) [Lp(a)] and oxidized phospholipids as potential interventions that target endothelial metabolism. It is important to note that all the metabolic states described in this manuscript are a reflection of the ‘activation state’ of the cells (i.e., proliferating cells, inflammatory cells) and should therefore be extrapolated into different contexts and disease pathologies with caution.

## Altered Metabolism As A Marker For Plaque Vulnerability

Although the molecular mechanisms that underlie cellular metabolic changes or rewiring are still being unraveled, the concept of an altered vascular metabolism has already been exploited for years. The tracer ^18^F-fluorodeoxyglucose (^18^F-FDG), a glucose analogue, is commonly used to image inflammatory cell activity non-invasively by Positron Emission Tomography (PET). It has been demonstrated that high arterial ^18^F-FDG uptake provides an independent predictor of future cardiovascular events. The tracer ^18^F-FDG is taken up via glucose transporters (GLUT) 1-and 3 by high-glucose demanding cells [19]. Subsequently, ^18^F-FDG is phosphorylated by hexokinases, after which ^18^F-FDG cannot be further metabolized resulting in intracellular accumulation that can be imaged [20,21]. Using FDG-PET in 17 patients with severe carotid stenosis, the group of Tawakol demonstrated that the PET signal significantly (*r* = 0.70; *p* < 0.0001) correlated to CD68-positive macrophages in the corresponding histologic sections [17]. In addition, when comparing FDG uptake with mean inflammation, the correlation with macrophage accumulation was stronger (*r* = 0.85; *p* < 0.0001), emphasizing how altered vascular metabolism, in particular increased glycolytic activity, can be utilized as a tool for distinguishing different plaque phenotypes. At the same time, deletion of the *Glut1* gene limits the enhanced glycolytic and mitochondrial activity of *ApoE^-/-^* hematopoietic stem and progenitor cells, attenuating the high-energy demand of these cells for proliferation and expansion and preventing the development of atherosclerosis [22]. Furthermore, when performing a targeted metabolomics approach on 159 carotid plaques from patients undergoing endarterectomy, Tomas and colleagues could distinguish symptomatic and highly vulnerable plaques (high-risk plaques) from low-risk plaques based on their metabolic profile [23]. Alongside high-risk plaques having increased secretion of the inflammatory mediators IL-6 (*p* < 0.0001), IL-8 (*p* < 0.0001), MCP-1 (*p* < 0.0001), IFNγ (*p* < 0.0001), IL-1β (*p* < 0.009), IL-18 (*p* < 0.0001) and MIP-1 β (*p* < 0.0001), these plaques could also be characterized by metabolites associated with altered energy metabolism. In addition to altered metabolite levels, mRNA levels of genes involved in glycolysis (*SLC2A3, HK2, HK3*) and the pentose-phosphate pathway (*PGD*) were significantly upregulated in the high-risk plaques. This data indicates a potential role for cellular metabolism, in this case glycolysis and the pentose-phosphate pathway (PPP), in determining plaque vulnerability. Although various cell types have been suggested to contribute to this observed altered cellular metabolism, including macrophages, monocytes, red blood cells, vascular smooth muscle cells and endothelial cells, the exact cellular source in these human plaques is not completely clear. In the following section, we will focus on the role of EC metabolism in regulating atherosclerosis.

## The Role Of Endothelial Metabolism In Angiogenesis, Inflammation And Atherogenesis

**Figure 1:**
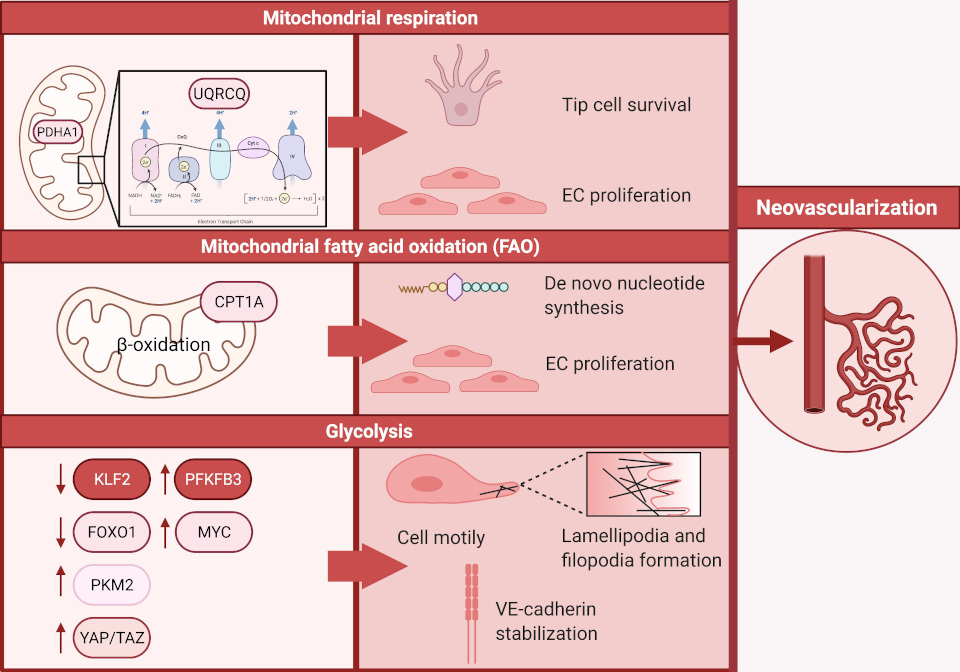
An overview demonstrating how glycolysis, mitochondrial respiration and fatty acid oxidation fuel neovascularization.

To regulate and sustain barrier function, blood flow, extravasation of leukocytes, macromolecules, solutes, fluids and hormones, rewiring of EC metabolism is essential. Approximately 80% of the ATP production in ECs in an in-vitro setting is derived from glycolysis, making glycolysis a predominant source of bioenergy [24–27]. In contrast to other cell types, ECs carry a low mitochondrial content accounting for approximately 2–5% of their cytoplasm, which suggests that in this context, mitochondrial respiration appears not the preferred route for ATP generation in ECs [28]. In the field of cancer biology, it has been established that ECs undergo a metabolic switch towards glycolysis to promote neovascularization, which facilitates tumor growth and metastasis [10,11]. Equivalently, neovascularization is pivotal in atherosclerotic lesions. These newly formed unstable and leaky vessels provide novel routes for the influx of pro-atherogenic lipoproteins, red blood cells, inflammatory cells and-mediators, and thereby contribute to plaque instability by forming thin-cap fibroatheromas that are more prone to rupture [8,9,29]. The formation of these new blood vessels rests on ECs specializing into leading tip cells that extend their filopodia and trailing stalk cells, which support extension of the sprouts by proliferation [30–32]. To date, a collection of studies describes the changes in EC metabolism that are essential in driving angiogenesis (neovascularization) and are extensively reviewed elsewhere [26,33,34]. In this review we aim to provide an overview of the candidates that are of interest in the context of atherosclerosis (a schematic overview can be found in Figure 1).

### Glycolysis

De Bock et al. demonstrated that in human umbilical venous endothelial cells (HUVECs), as well as arterial, lymphatic, and microvascular ECs glycolysis is the predominant bioenergetic pathway [35]. To investigate the role of glycolysis in ECs, they focused on the glycolytic enzyme 6-phosphofructo-2-kinase/fructose-2,6-biphosphatase 3 (PFKFB3). Upon knock-down of *PFKFB3*, an in-vitro sprouting assay showed a marked decrease in the number and length of sprouts (*p* < 0.05) [35]. In addition to these in-vitro results, mice with EC specific *Pfkfb3* deficiency demonstrated a decrease in branch points (*p* < 0.03) and distal sprouts with filopodia (*p* < 0.05) in a postnatal retina model. Knockdown of endothelial PFKFB3 resulted in smaller lamellipodia length (*p* < 0.05) and showed disoriented protrusions, resulting in hampered cells displacement and consequently impaired cell motility. Remodeling of the actin cytoskeleton and thus lamellipodia is crucial for EC migration. Combined, this indicates that PFKFB3-driven glycolysis regulates vessel sprouting via altering lamellipodia formation. In the context of atherosclerosis, it is important to note that in advanced human carotid plaques PFKFB3 expression was positively correlated with necrotic core area, indicating increased plaque vulnerability, whereas PFKFB3 was lower in the stable plaques having a thick fibrous cap [16]. This data illustrates that PFKFB3 expression appears to advance the progression of atherosclerosis and ultimately contributes to its clinical complications.

Additionally, the glycolytic enzyme pyruvate kinase M2 (PKM2) appears to be crucial for promoting angiogenesis [36,37]. PKM2 catalyzes the conversion of phosphoenolpyruvate to pyruvate while producing one ATP molecule. Silencing of *PKM2* expression in HUVECs resulted in decreased sprout formation and number of filopodia on tip cells [37]. Besides being localized in the cytoplasm, PKM2 has been found to localize together with VE-cadherin in the EC junctions as well as at F-actin-rich filopodia and lamellipodia of migrating ECs. *PKM2* silencing in HUVEC results in reduced junctional VE-cadherin expression. This coincides with an increase in discontinuous junctions, indicating that PKM2 is necessary for stabilization of EC junctions. Similarly, Jiang and colleagues demonstrated that PKM2 induces angiogenesis by upregulating glycolysis in human dermal lymphatic endothelial cells (HDLECs) [36]. Since neovascularization contributes to plaque instability, it would be interesting to investigate if atherosclerotic stimuli (e.g., oxidized LDL) are able to promote angiogenesis by induction of glycolysis and possible other metabolic pathways. This will provide the field with new insight into the mechanisms driving the progression of atherosclerosis and the factors that contribute to plaque instability.

### Mitochondrial Respiration

Yetkin-Arik and colleagues demonstrated that silencing the mitochondrial respiration enzyme pyruvate dehydrogenase E1 subunit alpha 1 (*PDHA1*) in HUVECs, resulted in an increased number of apoptotic tip cells and a decrease in proliferating cells [38]. This data underpins that besides glycolysis, mitochondrial respiration is also of importance in driving angiogenesis. Similarly, blocking pyruvate transport into mitochondria using 2-cyano-3-(1-phenyl-1H-indol-3-yl)-2-propenoic acid (UK5099), targeting the mitochondrial pyruvate carrier, resulted in a 30% reduction in the number of tip cells, indicating that mitochondrial respiration is essential for tip cell survival and EC proliferation. Similar effects were observed by the group of Diebold in HUVECs upon inhibition of the mitochondrial complex III using antimycin A and was attributed to decreases NAD^+^/NADH ratios [39]. The importance of mitochondrial respiration in angiogenesis is further highlighted by the observation that silencing *Pdha1* expression resulted in a 2.3-fold reduction in sprout length (*p* < 0.05) in in*-*vitro spheroid assays, followed by a decrease in branching points (*p* < 0.01) and total sprout length (*p* < 0.05) in the in-vivo chicken chorioallantoic-membrane photodynamic therapy (CAM-PDT) assay [38]. In line with these results, postnatal mouse retina angiogenesis assays demonstrated reduced radial expansion and branching in mice with endothelial-specific loss of ubiquinol-cytochrome C reductase complex III subunit VII (UQRCQ), which is crucial for mitochondrial respiration [39]. Furthermore, the group of Lapel reported diminished tubular formation of vasa vasorum ECs (VVECs) upon exposure to the OXPHOS inhibitors rotenone, oligomycin, and FCCP [40]. Collectively, these studies indicate a significant role for mitochondrial respiration in neovascularization. In conclusion, although the expression of glycolysis related markers has been established to be associated with increased plaque vulnerability, the importance of mitochondrial respiration in driving angiogenesis is nowadays becoming increasingly clear. It would be of interest to extrapolate these findings and assess the role of mitochondrial respiration in driving atherosclerosis in order to combat its progression.

### Fatty Acid Oxidation

Lastly, the function of mitochondrial fatty acid oxidation (FAO) in angiogenesis was studied by Schoors and colleagues [41]. Carnitine palmitoyltransferase 1 (CPT1) is a rate-limiting enzyme in FAO and is essential for beta oxidation of long chain fatty acids in the mitochondria. Silencing of the CPT1 isoform *CPT1A* in HUVECs resulted in a decrease in vessel sprout numbers and length (*p* < 0.0001), which was due to reduced EC proliferation (*p* < 0.001). Complementary to the human in-vitro data, endothelial specific loss of *Cpt1a* expression in mice resulted in a reduced number of branched points and diminished radial expansion in the postnatal retina due to decreased EC proliferation. Although loss of *CPTA1* did not affect ATP depletion or ADP/ATP ratio, *Cpt1a* was crucial for de novo nucleotide biosynthesis. Additionally, supplementation of *Cpt1a^KD^* ECs with acetate rescued nucleotide biosynthesis and the sprouting impairment. FAO has also been implicated in endothelial-to-mesenchymal transition (EndoMT). EndoMT-derived EC have been correlated with increased plaque burden [42] and decreased plaque stability in atherosclerosis [43]. The group of Finkel demonstrated a critical role for FAO, in particular for acetyl-CoA levels, in endothelial homeostasis both in-vitro and in-vivo [44]. Induction of EndoMT resulted in a decrease in acetyl-CoA levels. Supplementing acetate to the culture media was sufficient to inhibit EndoMT. Additionally, in adult *CPT1A* ^E-KO^ mice there was increased co-localization of endothelial and mesenchymal markers and increase in vascular permeability in the kidney, spleen and lung, thus demonstrating the importance of FAO in EndoMT. Comprehensively, these results display that besides glycolysis and mitochondrial respiration, FAO is a pathway to be recognized in facilitating neovascularization as well as endothelial homeostasis. To conclude, the importance of FAO should not be underestimated and future studies should shed more light on the potential importance of this metabolic pathway in the context of atherosclerosis.

## KLF2 AND FOXO1; Gatekeepers Of Ec Quiescence

Since endothelial cells form the inner lining of blood vessels, they are exposed to a force of laminar blood flow and shear stress. Disturbance of this blood flow can lead to disturbed shear stress, resulting in NF-κB-induced hypoxia-inducible factor 1α (HIF1α) transcription, promoting EC proliferation, activation and inflammation [45]. Besides the NF-κB-HIF1α signaling pathway, the AMPK/mTOR/ULK1-axis also have been demonstrated to be induced by shear stress [46]. This axis induces autophagy and thereby modulates vascular smooth muscle cells (VSMCs) phenotype. Similarly to VSMCs, autophagy is also essential in ECs for maintaining alignment [47].

### KLF2

In ECs the transcription factor Krüppel-like factor 2 (KLF2) promotes endothelial quiescence by upregulating anti-inflammatory and anti-thrombotic proteins and by downregulating pro-inflammatory and pro-thrombotic proteins. Upon exposure to laminar shear stress for 72 h, HUVECs induced KLF2 expression, which was accompanied by decreased glucose uptake and mitochondria per EC compared to static conditions [48]. This reduction in glycolysis was mediated by KLF2-induced downregulation of PFKFB3. This furthermore resulted in increased intracellular hyaluronan (HA) substrate availability and HA synthesis [49]. These results suggest that the KLF2-PFKFB3 axis has an important role in regulating EC metabolism and thereby altering the quiescent or activation state of the endothelium. It is therefore tempting to speculate that at sites where atherosclerotic plaques develop, characterized by disturbed or low-flow and low KLF2 expression, the inhibitory break on PFKFB3 expression is gone. This could lead to an increase in EC glycolysis, in turn fueling EC inflammation and facilitating atherogenesis. In the context of endothelial angiogenesis, overexpression of KLF2 significantly decreased sprout formation in in-vitro sprouting assays. Moreover, knocking-down KLF2 induced angiogenic sprout formation was subsequently blocked by the PFKFB3 inhibitor 3-(3-pyridinyl)-1-(4-pyridinyl)-2-propen-1-one (3PO) in the mouse aortic ring assay. Overall, these results suggest that the shear stress-KLF2-PBFKB3 axis has an important role in regulating EC metabolism under shear stress conditions, but can also affect plaque neovascularization.

### FOXO1

In addition to KLF2 as a gatekeeper for endothelial quiescence, the transcription factor forkhead box O1 (FOXO1) has been described as a metabolic checkpoint. Similarly to KLF2, FOXO1 is essential in regulating neovascularization [50]. Upon endothelial selective *Foxo1* deletion, *Foxo1* ^*i*EC–KO^ mice demonstrated a dense vasculature in the retina as a result of an increased number of ECs (*p* < 0.0001), filopodia (*p* < 0.0001) and enhanced vessel diameter (*p* < 0.001). *Foxo1* expression inhibits neovascularization by reducing glycolysis in ECs. Alongside reducing glycolytic activity, *Foxo1* expression also reduced oxidative phosphorylation, the formation of reactive oxygen species (ROS) and ATP levels. Lastly, gene set enrichment analysis (GSEA) established that *Foxo1* expressing cells repress MYC signature genes, which are involved in glycolysis and oxidative phosphorylation. Comprehensively, FOXO1 is a gatekeeper of EC quiescence by diminishing cellular metabolism via MYC inhibition.

### YAP-TAZ Signaling

The YAP/TAZ signaling pathway is also of importance in EC quiescence. Yes-associated protein (YAP) and transcriptional coactivator with PDZ-binding motif (TAZ) are activated by disturbed flow, resulting in translocation of the dephosphorylated form of YAP/TAZ into the nucleus, and thereby inducing the transcription of target genes [51]. This upregulation of YAP/TAZ target genes leads to enhanced proliferation and inflammation in ECs, demonstrated by enhanced retinoblastoma phosphorylation, and elevated expression of the adhesion molecules VCAM-1 and ICAM-1 which was accompanied with an increased adhesion of monocytes. This data indicates that regulation of YAP/TAZ is crucial for gatekeeping EC quiescence and consequently maintaining vascular homeostasis. Emerging evidence is accumulating that the YAP/TAZ pathway is intertwined with cellular metabolism [52–54]. The group of Enzo presented that YAP/TAZ, similarly to KLF2 and FOXO1, is regulated by glycolysis in MDA-MB-231 breast cancer cells [55]. In turn activation of the YAP/TAZ pathway in pulmonary arterial ECs has also been shown to modulate the metabolic enzyme glutaminase (GLS1), involved in glutaminolysis and glycolysis [56]. Collectively, these studies imply the existence of a YAP/TAZ metabolism positive feedback loop that could lead to the progression of atherosclerosis.

### CPT1A

Although ECs rely on glycolysis over OXPHOS for ATP, quiescent ECs have decreased glycolysis compared to proliferating ECs. Quiescent ECs are not hypometabolic, but they must adapt their metabolism to ensure redox balance and to maintain their baseline cellular processes. Recently, Kalucka et al. demonstrated that quiescent HUVECs also upregulate FAO up to 3- to 4-fold more than in proliferating HUVECs [57]. Additionally, when becoming quiescent, glycolytic flux and glucose consumption were reduced. However, unlike in proliferating ECs, this upregulation of FAO was vital for redox homeostasis rather than nucleotide synthesis. Importantly, conditional inactivation of *CPT1A* in ECs in-vivo caused oxidative stress and an upregulation of genes involved in redox homeostasis. Given the high oxygen environment quiescent ECs find themselves in, redox balance is likely a vital process in maintaining endothelial homeostasis and quiescence.

## The Rise Of Mirnas

Over the years microRNAs (miRNAs) have been emerging as significant regulators in atherosclerosis with novel functions being discovered regularly. The current status of miRNAs and their therapeutic potential in atherosclerosis have been extensively discussed by Feinburg and Moore [58]. This review aims to focus on miRNAs that are specifically involved in cellular metabolism in the context of atherosclerosis.

### Inflammation

Oxidized phospholipids (OxPLs) are also known as Danger Associated Molecular Patterns (DAMPs) that can be carried by lipoprotein(a) and oxLDL, resulting in accumulation in atherosclerotic lesions [59]. Here oxPLs can induce an inflammatory response and thereby aggravate disease progression [60]. Upon exposure of HUVECs to 30 μg/mL oxidized phospholipid 1-palmitoyl-2-arachidonoyl-sn-glycero-3-phosphocholine (oxPAPC), the transcription factor Nuclear factor erythroid 2-Related Factor 2 (NRF2) was activated, leading to the expression of the miR-106b~25 cluster member, miR-93 [61]. In addition to increasing proliferation (*p* < 0.01), miR-93 expression resulted in an enhanced glycolytic rate (*p* < 0.05) and capacity (*p* < 0.05). Using RNAseq and qPCR analysis it was demonstrated that this miR-93-induced glycolysis was mediated by upregulation of the glycolytic enzyme PFKFB3 and downregulation of the transcription factors KLF2 and FOXO-1. Furthermore, MYC was observed to be a direct target of miR-93, indicating that miR-93 activates the FOXO1-MYC axis. This data combined with the observation that oxPLs induce inflammation indicates that miR-93 potentially increases inflammation by enhancing endothelial glycolysis. However, further research is necessary to further investigate the intricate connection between miR-93, inflammation and glycolysis.

### Pulmonary Arterial Hypertension

Lastly, the effect of anomalous miRNA expression in cardiovascular disease was highlighted in-vivo by Caruso and colleagues. They demonstrated that pulmonary vascular and circulating progenitor ECs derived from patients with pulmonary arterial hypertension (PAH) downregulated miR-124 [62]. This downregulation of miRNA-124 resulted in increased expression of the target protein, splicing factor polypyrimidine-tract-binding protein (PTBP1), alongside upregulation of the glycolytic enzyme pyruvate kinase M2 (PKM2). Notably, supplementation of miR-124 in PAH blood outgrowth ECs (BOECs) normalized the expression of glycolysis related genes by downregulating *LDHA*, *PDK1*, *PDK2* and *MCT1* expression, as well as increasing PKM1 expression. This normalization in gene expression resulted in functional changes demonstrated by a diminished glycolytic flux and lactate secretion. Furthermore, MiR-124 supplementation restored mitochondrial activity to basal levels. Comprehensively, these results indicate that miR-124 supplementation could be a feasible strategy to target altered endothelial metabolism, making miR-124 a promising drug target for future pharmaceutical interventions in atherosclerosis.

## Inflammaging And Senescence Are Inextricably Linked To Cellular Metabolism

Atherosclerosis is a multifactorial process that drives cardiovascular disease, and has been associated with several risk factors, including age. Especially, in the Western world we are being confronted with a growing aging population, which increases the risk of major adverse cardiovascular events (MACE) [63,64]. To be able to treat this expanding patient population, it is necessary to understand how aging affects the atherosclerotic process. One of the key hallmarks of ageing is the growing number of cells that turn senescent, thus an increase in cells that are in proliferation arrest. Recently, Sabbatinelli and colleagues extensively reviewed the metabolic rewiring that senescent ECs undergo in order to sustain their activities. This rewiring is characterized by an even higher dependency on glycolysis, the production of ROS, a decrease in nitric oxide (NO) production and induction of pro-inflammatory processes [65], demonstrating a metabolism-senescence-inflammation axis in aging individuals.

During aging, mitochondrial function declines, thereby contributing to the acceleration of atherosclerosis [66]. Using wild-type mice in a low cholesterol environment, Tyrrell and colleagues demonstrated that, along with an increase in mitochondrial dysfunction, aged mice also exhibit elevated levels of IL-6 within the aorta [66]. Mitochondrial damage-associated molecular patterns expressed by dysfunctional mitochondria activate the TLR-9-MyD88 axis, resulting in the production of pro-inflammatory cytokines including IL-6. In turn IL-6, can further aggravate mitochondrial dysfunction, suggesting a positive feedback loop within the aorta of aging mice. Furthermore, this enhanced mitochondrial dysfunction was also characterized by an increase of mitophagy, which is the degradation of mitochondria by autophagy. Recently, encouraging evidence is accumulating about the potential of targeting mitochondrial function as strategy for maintaining vascular function [67]. By supplementing the mitochondrial-targeted antioxidant MitoQ in aged (approximately 27 months) mice, it has been demonstrated that targeting mitochondrial fitness reduced the production of mitochondrial derived ROS and restored endothelium-dependent dilation [68]. These promising pre-clinical data were confirmed in a randomized, placebo-controlled, double-blind, crossover design study with healthy older adults (60–79 years) [69]. Individuals receiving 6 weeks MitoQ supplementation exhibited a 42% higher NO-dependent brachial artery flow-mediated dilation (BAFMD), which is a measurement for endothelium-dependent dilation (EDD). Interestingly, oxLDL levels in the plasma of individuals treated with MitoQ were decreased compared to the individuals receiving placebo.

Besides changes on a cellular level, aging individuals are also confronted with increased stiffness of the large arteries. There are several mechanisms underlying this arterial stiffness, which have been extensively reviewed by several groups [70–72]. Vessel stiffening has been shown to increase endothelial permeability [73]. In tumor vasculature, PFKFB3 has been implicated in vessel destabilization due to VE-cadherin internalization [11]. Inhibiting PFKFB3 led to increased barrier integrity, due to increased expression of VE-cadherin at the membrane. This suggests that increased PFKFB3 activity can lead to vessel destabilization and increased endothelial permeability. Additionally, vessel stiffness and shear stress-mediated EC alignment are also linked. Bovine aortic ECs cultured on hydrogels mimicking older, stiffer vessels form less tight junctions after 24-hour exposure to fluid shear stress compared to ECs cultured on hydrogels mimicking younger vessels [74]. To this end, loss of eNOS activity due to disturbed shear stress, results in lower NO production, an increase in blood pressure and thereby an increased likelihood of vessel wall injury [2], a process that is accelerated in aging individuals and hallmarked by increased blood vessel stiffening.

## The Metabolic-Inflammatory Axis In Endothelial Cells

### Trained Immunity

In the context of trained immunity, the influence of inflammation on rewiring metabolism in monocytes and the subsequent sustained inflammatory effects have been well documented [59,75,76]. Accumulating evidence suggests that circulating lipoproteins elicit trained immunity in monocytes [59,77]. The pro-atherogenic lipoprotein oxLDL has been recognized to induce trained immunity in primary monocytes demonstrated by an enhanced secretion of IL-6, TNFα, IL-8 and MCP-1, thereby contributing to the persistent low-grade inflammation observed in atherosclerosis [77]. Recently it has been demonstrated that metabolic reprogramming is required for oxLDL induced trained immunity [78]. Using extracellular flux analysis Keating and colleagues observed an increased ECAR (*p* < 0.05) in oxLDL trained macrophages, that was accompanied by an upregulation of the glycolytic enzyme PFKFB3 (*p* < 0.05). Along with this increase in ECAR, there was also an enhanced OCR (*p* < 0.05). Overall, these results indicate a metabolic switch upon oxLDL induced training. This was further validated by demonstrating that the susceptibility for trained immunity in individuals was associated with genetic variations in glycolytic genes, including *PFKFB3*, *PFKP* and *HK1*.

### Endothelial Cells

Increasing evidence suggests that a similar metabolism-inflammation-axis may also exist in endothelial cells [4,11,57]. As previously described, an endothelial specific knockout of the rate-limiting FOA enzyme *Cpt1a* in mice resulted in a reduction in neovascularization as well as an effect on inflammation [57]. At 24 hours after LPS injection, *Cpt1a* ^ΔEC^ mice had elevated serum levels of the pro-inflammatory cytokines IFNγ, TNFα, IL-5, IL-17 and IL-23. Furthermore, upon treatment with IL-1β and TNFα ECs demonstrate an increase in glycolysis and *Pfkfb3* gene expression [11]. Interestingly, PFKFB3 inhibition using 3PO diminished the response to cytokine activation by reducing NF-κB signaling, indicating a complex interaction between by PFKFB3 mediated glycolysis and inflammatory pathways. In line with these results, Schnitzler and colleagues demonstrated a role for PFKFB3 in Lp(a) induced inflammation [4]. OxPLs carried by Lp(a) elicit a pro-inflammatory response in human arterial ECs (HAECs) [4,59]. Upon stimulation with Lp(a), HAECs secreted the pro-inflammatory cytokines IL-6, IL-8, and MCP-1, with a concomitant increase in the adhesion molecules E-cadherin, ICAM-1 and VCAM-1 [4]. Furthermore, it was established that oxPL-driven inflammation was facilitated by an increase in PFKFB3-mediated glycolysis, indicating the presence of a metabolic-inflammatory-axis in ECs. Interestingly, inhibition of PFKFB3 activity using the inhibitor PFK158 resulted in the nullification of atherogenesis (as attested by reduction in cytokine secretion and leukocyte adhesion molecule expression) and as a functional consequence, reduced monocyte migration through the endothelium. To further validate the significance of oxPLs in driving inflammation and atherosclerosis in vivo, transgenic mice expressing a single chain variable fragment of E06 (E06-scFv) were generated on a *Ldlr^-/-^* background [79]. *Ldlr^-/-^*/E06-scFv mice on a high cholesterol diet (HCD) demonstrated a significant reduction in lesion area of the entire aorta at 4 (57%; *p* = 0.016), 7 (34%; *p* = 0.045) and 12 (28%; *p* = 0.012) months compared to *Ldlr^-/-^* on a HCD. Along with this reduction in lesion size, *Ldlr^-/-^*/E06-scFv mice demonstrated diminished systematic inflammation presented by a 32% reduction (*p* = 0.016) in plasma serum amyloid A (SAA). Collectively, the reduction of these pro-atherogenic processes resulted in *Ldlr^-/-^*/E06-scFv mice having a prolonged life (Log-rank *p* = 0.016) compared to *Ldlr^-/-^* mice measured over 15 months This data implies a pivotal role for both oxPLs as well the existence of 2a metabolic-inflammatory axis in driving atherogenesis.

## Potential Therapeutic Interventions Targeting Vascular Metabolism In Atherosclerosis

### Current Treatment; Statins and Their Effect on Endothelial Metabolism

Statins have been used as a therapeutic strategy against cardiovascular disease for decades. Besides their primary mechanism of action, lowering LDL-C levels, statins have been demonstrated to exhibit pleiotropic effects which decrease cardiovascular burden independently of LDL-C lowering [80]. Here, we will briefly focus on the pleiotropic effects related to endothelial metabolism. Altun and colleagues demonstrated that atorvastatin (40 mg/day) treatment in patients with acute coronary syndrome improved the flow-mediated vasodilation, which was accompanied with a decrease in the adhesion molecules E-selectin and sICAM-1 and the inflammatory marker C-reactive protein (CRP) [81]. These studies indicate that statins have a direct effect on endothelial function. Various in-vitro and in-vivo studies attribute this improvement of endothelial function to statin-induced NO production by AMPK mediated upregulation of eNOS activity, indicating a role for statins in the endothelial-NO-inflammatory-axis [82–86].

### A Novel Approach; Directly Targeting Vascular Metabolism

Altered endothelial metabolism is inextricably linked to atherosclerosis, especially PFKFB3 has been illustrated as a key regulator of glycolysis in ECs, and could therefore be a potential drug target (Table 1). In cancer research, PFKFB3 has already been extensively studied as a target for therapeutic intervention. Cantelmo et al. demonstrated that tumor ECs are characterized by a significant increase in transcription of glycolysis related genes (FDR-adjusted *p* = 0.023) [11], including PFKFB3, compared to normal endothelial cells. In addition, liquid chromatography-mass spectrometry (LC-MS) analysis demonstrated a rise in glycolysis related metabolites as well as an increase in glucose consumption, lactate excretion and glycolytic flux. This data emphases the importance of glycolysis in tumor endothelial cells, an effect that could be reversed by inhibition of PFKFB3 with 3PO. While treatment had no effect on cancer cells, PFKFB3 inhibition did result in a significant reduction in lung metastasis (*p* < 0.05) and was accompanied by tumor vessel normalization, demonstrated by increased vessel lumen size, increased perfusion, a decrease in hypoxia markers, an upregulation of VE-cadherin and an enhanced recruitment of pericytes. Normalization of the vasculature is not merely of importance in cancer biology, but is also crucial for overcoming the endothelial dysfunction and loss of barrier function observed in atherosclerosis. Therefore, it would be of interest to determine the effect of PFKFB3 inhibition in ECs that were exposed to atherogenic stimuli such as oxLDL or Lp(a).

Along with restoring vascular homeostasis, 3PO has also been shown to be effective in reducing pathological angiogenesis in ocular and inflammatory models [87]. Previous studies have also shown that neovascularization in atherosclerotic plaques contributes to increased plaque instability [8,9]. Therefore, the observations that 3PO can reduce pathological angiogenesis could be beneficial for plaque stability and consequently the risk of MACE. Keating et al. have demonstrated that oxLDL induces trained immunity in monocytes by increasing glycolysis and oxygen consumption [78]. Using genotype information and PBMCs from 119 healthy volunteers from the Human Functional Genomics Projects, it has been demonstrated that genetic variation in the enzymes involved in glycolysis determine to which degree individuals are susceptible to oxLDL induced trained immunity. Therefore, 3PO was used to investigate if targeting glycolysis could reduce oxLDL induced trained immunity. Accordingly, co-incubation of 3PO and oxLDL resulted in a dose dependent decrease in the production of the inflammatory cytokines TNFα and IL-6.

Recently, Poels et al. investigated the effect of PFKFB3 inhibition by PFK158 (a derivative of 3PO) in a murine in-vivo atherosclerosis model. *Ldlr^-/-^* mice on a high fat diet were treated with PFK158 for 5 weeks. Plaques from the PFK158 treated group had less incidence of fibrous cap atheroma (*p* < 0.05), accompanied by a significant reduction in necrotic core area (*p* < 0.05) and apoptotic cell (TUNEL) staining area (*p* < 0.005) [16]. Moreover, there was increase in vascular smooth muscle content (*p* < 0.005). And thickening of the fibrous cap area (*p* < 0.05). Altogether, these aspects contribute to plaque stability, as indicated by the significant increase in stability index area (*p* < 0.05) of the PFK158 treated group.

Taken together, pharmacological therapeutic interventions directly or in-directly targeting vascular metabolism appear to be beneficial by increasing plaque stability, diminishing inflammation and reducing neovascularization in in-vitro and/or in-vivo models.as summarized in Table 1.

### Targeting Atherogenic Stimuli That Induce Altered Vascular Metabolism

In parallel to directly targeting vascular metabolism, it would be advantageous to reduce the atherogenic stimuli that induce metabolic reprogramming in ECs in the first place. As described previously, Lp(a) induces vascular glycolysis, thereby initiating a pro-inflammatory endothelial phenotype that facilitates leukocyte extraversion [4]. This Lp(a)-induced, glycolysis mediated pro-atherosclerotic phenotype could be reduced by decreasing the high elevated Lp(a) levels or using specific antibodies targeting oxidized phospholipids bound to the apo(a) tail. Recently, a phase 3 apo(a) antisense clinical trial has been initiated to assess the impact of antisense oligonucleotides (ASOs) targeting apolipoprotein(a) (AKCEA-APO(a)-LRx) on MACE in patients with CVD (ClinicalTrials.gov Identifier: NCT04023552). Previous phase 1 and 2 clinical trials using the AKCEA-APO(a)-LRx ASO significantly reduced Lp(a) levels in patients with cardiovascular disease and elevated Lp(a) levels with a favorable safety and tolerability profile [88]. Stiekema and colleagues established that potent Lp(a) lowering by AKCEA-APO(a)-LRx diminishes the pro-inflammatory phenotype of circulating monocytes observed in patients with elevated Lp(a) levels [89]. Along with reduced transcripts of pro-inflammatory genes, potent Lp(a) lowering resulted in a reduction of the surface expression of CCR2 (*p* = 0.0479), CX3CR1 (*p* = 0.0005) and TLR2 (*p* = 0.0024) accompanied with a decrease in trans-endothelial migration (*p* = 0.0002). Overall, Lp(a) lowering induced transcriptional and functional changes in circulating monocytes.

Alongside lowering Lp(a) levels by ASOs, monoclonal antibody treatments have been developed to inhibit the atherogenic effect of oxidized phospholipids carried by lipoproteins, including Lp(a) and oxLDL, where, targeting these oxPLs appears to be a suitable option to reduce endothelial inflammation. The monoclonal antibody E06, binding to oxidized phospholipids diminished the Lp(a)-induced expression of the adhesion molecules ICAM-1 and VCAM-1, and inflammatory markers IL-6 and IL-8. This reduction in adhesion molecule expression also translates into a decrease in CD14^+^ + monocyte adhesion (*p* = 0.0324) and transmigration (*p* = 0.0095) through the endothelial layer. Overall, blocking oxPLs by E06 diminishes Lp(a)-induced inflammation and activation of ECs.

## Conclusions, Limitations And Future Perspectives

### Conclusions

In this review, we discussed the impact of vascular metabolism in atherosclerosis and its progression along with shedding some light on the potential of targeting these altered metabolic pathways. Although there are several treatment options on the market for slowing the progression of atherosclerosis, CVD remains the number one cause of death worldwide and is still increasing, in part due to our rapidly growing aging population [63,64]. Aging as well as the exposure to atherosclerotic stimuli are able to rewire cellular metabolism in the vasculature [2,4,11,65,66,78]. This metabolic rewiring in ECs results in endothelial activation, consequently inducing neovascularization and creating a pro-inflammatory environment that facilitates leukocyte extravasation [3–9]. Both processes drive the progression of atherosclerosis and contribute to plaque instability, illustrating the importance of these pathways [8,9,29].

### Limitations and Future Perspectives

As stated previously multiple studies showed the beneficial therapeutic effect of targeting altered EC metabolism in several atherosclerosis models (Table 1). However, currently most of these interventions have not entered clinical trials. In order to be able to translate these experimental findings into the clinical arena, new scientific advances in the field of vascular and immunometabolism are warranted. For instance, most in-vitro studies discussed in this review were performed with HUVECs. HUVECs are a preferred endothelial model, since they are easily to retrieve and have a high proliferation rate [90,91]. Additionally, HUVECs can migrate and invade, making them suitable for several angiogenesis and transmigration assays [90,92]. However, HUVECs do not fully recapitulate the vascular bed affected in atherosclerosis [91]. It is therefore important to take this into account when extrapolating the data into the context of their respective disease etiology. Therefore, future studies could take the different disease pathologies into account as well as the tissue of interest and adapt their cell lines accordingly. To illustrate, HAECs could be one of the preferred cell-types when studying atherogenesis [93,94]. Alongside utilizing the appropriate cell lines, the field of vascular metabolism could also benefit from the use of advanced in-vitro models, such as organ-on-a-chip technologies, co-culture systems and human induced pluripotent stem cells. These in-vitro models provide a platform to mimic the complex multifactorial aspects of the vasculature, making the results accessible to translate towards the clinic [95].

Besides exposure to atherogenic stimuli, the role of aging is significant for the elevated dependency on glycolysis, increased mitochondrial dysfunction, ROS production and inflammation as well as the decrease in NO production [65,66]. However, the plasticity of EC metabolism in aging individuals has been discussed to a lesser extent. This generates the question whether novel therapeutic interventions targeting metabolism can switch EC metabolism to their original state, thereby restoring EC phenotype and consequently vascular homeostasis. Implementing these outstanding questions in future research in the field of vascular metabolism will help move the field forward.

In this review we described various metabolic pathways that can be altered in ECs, where the glycolytic pathway being the one being that has been extensively investigated and therefore mostly discussed. PFKFB3 inhibition have been described in the context of cancer by several landmark studies by the group of Carmeliet [11,26,78]. In the context of atherogenesis, inhibition of PFKFB3 showed promising results in the first in-vitro studies as well as in-vivo studies, demonstrating the therapeutic potential of targeting of vascular metabolism as a therapeutic strategy to combat atherosclerosis [4,16].

However, it is important to realize that—just like any other cellular processes—the adaptation of metabolism is, amongst others, dependent on time, spatial localization, their ‘cellular state’ (i.e., quiescent, proliferative, activated/inflamed) but also on the available energy supply and demand [96]. This makes extrapolation of the metabolic state of ECs from one disease to another extremely challenging. While some data suggests that EC activation and inflammatory responses precede the observed increase in glycolysis [18], the opposite could also be true for example in diabetic patients. Here, the sustained glucose supply and increased glycolytic flux by itself may also cause EC activation and inflammation [97,98]. Therefore, further unraveling of the metabolic-inflammatory axis in ECs in the proper (patho)physiological context is necessary to provide this and other exciting fields with detailed insight in which metabolic regulators could be targeted to reduce the atherosclerotic burden.

## Figures and Tables

**Table 1:** Overview of potential strategies for targeting altered metabolism in atherosclerosis discussed in this review.

Therapy	Target	Experimental model	Therapeutic target/goal	Reference
miR-124 Supplementation	PTBP1 and PKM2	blood outgrowth endothelial cells from patients with pulmonary arterial hypertension	Restoring mitochondrial activity Normalizing glycolysis	[62]
Supplementation with the anti-oxidant MitoQ	Mitochondria	Aged mice	Reduction of ROS Restoring endothelium-dependent dilation	[68]
Supplementation with the anti-oxidant MitoQ	Mitochondria	healthy older adults (60–79 years)	Restoring endothelium dependent dilation	[69]
Small molecule 3PO	PFKFB3	Orthotopic pancreatic and B16-F10 melanoma tumour models	Normalization of the vasculature	[11]
Small molecule 3PO	PFKFB3	in vitro HUVEC spheroid models in vivo zebrafish embryos and postnatal mouse retinas	Reducing neovascularization	[87]
Small molecule 3PO	PFKFB3	Peripheral blood mononuclear cells	Reducing inflammation	[78]
Specific inhibitor PFK158	PFKFB3	LDLr^-/-^ mice on a high fat diet	Increasing atherosclerotic plaque stability	[16]
Atorvastatin (40 mg/day)	eNOS activity	Patients with acute coronary syndrome	Increasing FMD, decreasing E-selectin, sICAM and CRP	[81]
AKCEA-APO(a)-LRx	apolipoprotein(a)	Phase 1 and 2 clinical trials	Lowering Lp(a), Reducing the pro-inflammatory phenotype of circulating monocytes	[88,89]
Monoclonal antibody E06	Oxidized phospholipids	Human arterial endothelial cells	Decreasing Lp(a)-induced EC inflammation and activation	[4]

## Abbreviations

^18^F-FDG: ^18^F-fluorodeoxyglucose
3PO: 3-(3-pyridinyl)-1-(4-pyridinyl)-2-propen-1-one
BAFMD: Brachial artery flow-mediated dilation
BOEC: Blood outgrowth endothelial cell
BrdU: 5-bromo-2’-deoxyuridine
CAM-PDT: Chicken chorioallantoic-membrane photodynamic therapy
CPT1: Carnitine palmitoyltransferase 1
CRP: C-reactive protein
EC: Endothelial cell
ECAR: Extracellular acidification rate
EDD: Endothelium-dependent dilation
FAO: Fatty acid oxidation
FMD: Flow-mediated vasodilation
FOXO1: Forkhead box O1
GLUT: Glucose transporter
GSEA: Gene set enrichment analysis
GSK3A: Glycogen synthase kinase-3 alpha
HAEC: Human arterial endothelial cell
HA: Hyaluronan
HIF1α: Hypoxia-inducible factor 1α
HMVECs: Human microvascular endothelial cells
HUVECs: Human umbilical venous endothelial cells
KLF2: Krüppel-like factor 2
LDL: Low-density lipoprotein
Lp(a): Lipoprotein(a)
LC-MS: Liquid chromatography-mass spectrometry
MACE: Major adverse cardiovascular events
NO: Nitric oxide
NRF2: Nuclear factor erythroid 2-Related Factor 2
OCR: Oxygen consumption rate
OxPL: Oxidized phospholipid
PAH: Pulmonary arterial hypertension
PAPC: 1-palmitoyl-2-arachidonoyl-sn-glycero-3-phosphocholine
PDHA1: Pyruvate dehydrogenase E1 subunit alpha 1
PET: Positron emission tomography
PFKFB3: 6-phosphofructo-2-kinase/fructose-2,6-biphosphatase 3
pHH3: Phospho-histone H3
PKM2: Pyruvate kinase M2
PTBP1: Splicing factor polypyrimidine-tract-binding protein
ROS: Reactive oxygen species
TEM: Transendothelial migration
UQRCQ: Ubiquinol-cytochrome C reductase complex III subunit VII
VSMC: Vascular smooth muscle cell
VVEC: Vasa vasorum endothelial cell
YAP: Yes-associated protein
TAZ: Transcriptional coactivator with PDZ-binding motif
